# Development and Application of a Comprehensive Measure of Access to Health Services to Examine COVID-19 Health Disparities

**DOI:** 10.3390/healthcare11030354

**Published:** 2023-01-26

**Authors:** Fathima Wakeel, Haiyan Jia, Lifang He, Karmel S. Shehadeh, Lucy E. Napper

**Affiliations:** 1 Department of Community and Population Health, College of Health, Lehigh University, Bethlehem, PA 18015, USA; 2 Department of Journalism & Communication, College of Arts and Sciences, Lehigh University, Bethlehem, PA 18015, USA; 3 Department of Computer Science & Engineering, P.C. Rossin College of Engineering and Applied Science, Lehigh University, Bethlehem, PA 18015, USA; 4 Department of Industrial and Systems Engineering, P.C. Rossin College of Engineering and Applied Science, Lehigh University, Bethlehem, PA 18015, USA; 5 Department of Psychology, Health, Medicine, and Society Program, College of Arts and Sciences, Lehigh University, Bethlehem, PA 18015, USA

**Keywords:** access to health services, access to healthcare, access to community resources, health disparities, COVID-19 pandemic

## Abstract

Research on access to health services during the COVID-19 pandemic is limited, and the conceptualization of access has not typically included access to community resources. We developed and tested an access-to-health-services measure and examined disparities in access among individuals in the U.S. during the pandemic. Data are from a U.S. sample of 1491 respondents who completed an online survey in August 2021. Linear regression models assessed the relationships between the access-to-health-services-measure components, including impact on access to medicine and medical equipment, impact on access to healthcare visits, and confidence in accessing community resources, and predictor variables, including sociodemographic- and health-related factors. Disparities in access to healthcare during the pandemic were associated with sociodemographic characteristics (i.e., race, gender, and age) and health-related characteristics (i.e., chronic illness, mental health condition, and disability). Factors such as race, gender, income, and age were associated with individuals’ degree of confidence in accessing community services. Our study presents a new access-to-health-services measure, sheds light on which populations may be most vulnerable to experiencing reduced access to health services, and informs the development of programmatic interventions to address the salient needs of these populations.

## 1. Introduction

Access to healthcare has been a prevailing problem in the United States (U.S.). A 2013 report from the World Health Organization [1] attributed “at least some of the problems with United States performance with respect to health outcomes” as “a result of poor access to care” [1] (p.321). Further, there has been widespread evidence of healthcare disparities related to sociodemographic characteristics. Healthcare disparities among racial and ethnic groups have a long history and are still prevalent in the U.S. Nationally, the magnitude of racial and ethnic disparity depends significantly on the state and the racial/ethnic group [2]. High-risk patients in historically excluded communities, e.g., women and African American HIV patients [3], as well as children with certain racial and ethnic backgrounds [4] and in immigration families [5], face more significant challenges, such as care availability and health insurance coverage. Gender is another marker of social and economic vulnerability, with women being particularly vulnerable to income inadequacy and caregiving responsibilities [6]. Socioeconomic status [7,8], race and ethnicity [9], and age [10] contribute to the healthcare disparities observed among women. In addition to women, gender and sexual minorities also face substantial disparities. A 2019 review [11] recognized healthcare disparities for the more than 10 million lesbian, gay, bisexual, and transgender (LGBTQ+) persons living in the U.S.

Global pandemics tend to exacerbate existing health disparities and have contributed to increased morbidity and mortality in underserved populations. For instance, during the H1N1 influenza pandemic, the risk of exposure was significantly related to race and ethnicity [12]. The COVID-19 pandemic is a public health emergency that has highlighted and worsened existing health inequities in the U.S. [13]. Early research has already shown that telemedicine use contributes to widening health disparities, as underserved populations, including older individuals, some racial and ethnic minorities, non-English speaking individuals, and those who use Medicaid insurance, have limited access to telemedicine [14,15].

The literature has defined *access to health services* in a myriad of ways. The Institute of Medicine has broadly defined access as “the timely use of personal health services to achieve the best health outcomes” [16] (p. 4). There has been debate about whether access entails the availability of services that individuals can feasibly obtain or if it also includes the actual utilization of services [17]. Many researchers, however, have incorporated the utilization of needed services into conceptualizing access to health services [18,19]. During the COVID-19 pandemic, the World Health Organization [20] developed a report identifying the essential health services that were most commonly disrupted during the pandemic, including routine immunization services, noncommunicable disease diagnosis and treatment, family planning and contraception, treatment for mental health disorders, antenatal care, and cancer diagnosis and treatment. Another critical aspect of access that has been examined is *access to medicines* [21,22].

Despite the widespread focus on the conceptualization and measurement of access to health services in the extant literature, the construct of access has not adequately incorporated the social determinants of health, such as access to food, housing, and financial resources, that play an instrumental role in shaping one’s health trajectory [23]. Moreover, *access to community resources* is a significant concern, especially during a global pandemic, as individuals rely on these resources and community support to deal with unprecedented challenges and safeguard their wellbeing. However, there has been little research on access to community resources during the pandemic. Some evidence suggests that access to community food banks and food insecurity has been an issue for many racial and ethnic minority families during the pandemic, partly because this population faced a higher unemployment rate [24,25].

Research on access to health services during the COVID-19 pandemic, especially on a broader scale, is limited [14,15]. Further, the conceptualization of access has not typically included access to community resources. To fill these knowledge gaps, we developed and tested a novel access-to-health-services measure during the COVID-19 pandemic using nationwide data inclusive of a wide range of sociodemographic and health characteristics. Specifically, our study aims to: (1) describe the access to health services among individuals in the U.S. during the pandemic using our proposed access-to-health-services measure, which includes the components of *access to medicine and medical equipment*, *access to healthcare visits*, and *access to community resources*, and (2) examine the sociodemographic- and health-related disparities in access to health services during the pandemic. Our study contributes a more comprehensive approach to conceptualizing access to health services and generates insights that inform public health policy and programs for future COVID-19 resurgence and emergent health crises.

## 2. Materials and Methods

### 2.1. Sample and Procedures

Using an online survey, we assessed access to health services, including access to healthcare and confidence in access to community resources, and examined how various socioeconomic factors and health-related variables impacted access to healthcare and community resources. The 30-min survey was administered among a nationwide sample of 1500 respondents on Prolific, a web-based survey recruitment platform [26], in August 2021. *Prolific* provided our Qualtrics survey link to eligible respondents on their platform. These individuals were directed to our survey if they met our eligibility criteria (i.e., US residents who were at least 18) and provided informed consent after reading the consent statement. Each respondent received a USD 5 incentive for participation. Previous research [27,28,29,30] has attested to the comparatively high data quality of *Prolific* in terms of its research ethics, user honesty and reliability, and demographic diversity and representativeness. The Lehigh University Institutional Review Board approved this observational study. After removing responses with missing values for key variables, we conducted our analysis based on 1491 responses. 

### 2.2. Measures

#### 2.2.1. Access to Health Services

To develop the access-to-health-services measure and understand the impact of the COVID-19 pandemic on access, items were generated by reviewing the literature on healthcare access [31,32,33,34] and evaluated by a multidisciplinary team of researchers. Specifically, with regard to *access to healthcare*, we asked: “How has the COVID-19 pandemic impacted your ability to manage your health?” Respondents were asked to indicate if they experienced any of the following impacts on their access to healthcare: obtaining equipment or supplies, obtaining medications, canceling or rescheduling a procedure or surgery, office closure, postponing a healthcare appointment, and skipping a healthcare appointment.

Survey data from the 1491 participants were used to conduct a principal components analysis (PCA) to validate and assess the healthcare-access items. Two factors underlying the “access-to-healthcare” variables were identified: (1) *access to medicine and medical equipment* (comprising the following two items: obtaining equipment or supplies and obtaining medications; range of 0 to 2); and (2) *access to healthcare visits* (including the following three items: office closure, postponing a healthcare appointment, and skipping a healthcare appointment; range of 0 to 3). Using the normalized data, the factor analysis (Table 1) with varimax rotation yielded all factor loadings except one over 0.6 and cross-loadings below 0.4. The low-loading item was removed, and the two identified indices were used in further analyses as components of the access-to-health-services measure. The access to medicine and medical equipment and access to healthcare visits indices had acceptable reliability with Cronbach’s alphas of 0.65 and 0.65, respectively.

We also incorporated access to community resources during the pandemic into the access-to-health-services measure by using the question: “How confident do you feel about your ability to access the following community resources if needed?” Respondents rated on a scale of 1–5, with higher numbers indicating greater confidence, their confidence in accessing the following community resources: food access support, housing support, domestic violence resources, mental health resources, substance misuse resources, parenting/family support services, LGBTQ+ support, and spiritual/religious resources. The selection of these community resources was based on key social determinants of health, mental, and spiritual health and family health resources. Responses to these items were recoded to range from 0 to 4. PCA indicated that the items were unidimensional. Additionally, a reliability test showed high internal consistency (Cronbach’s alpha = 0.89). Therefore, the items were summed to create the *access to community resources* index, which ranged from 0 to 32. Table 2 lists all the items of the three components of the access-to-health-services measure.

#### 2.2.2. Sociodemographic Variables

We examined the following sociodemographic correlates of access to health services during the pandemic:

##### Race/Ethnicity

This category included American Indian or Alaska Native (e.g., Navajo Nation, Blackfeet Tribe, Mayan, Aztec, Native Village of Barrow Inupiat Traditional Government, Nome Eskimo Community); Asian (e.g., Chinese, Filipino, Asian Indian, Vietnamese, Korean, Japanese); Black or African American (e.g., Jamaican, Haitian, Nigerian, Ethiopian, Somalian); Hispanic, Latino, or Spanish Origin (e.g., Mexican or Mexican American, Puerto Rican, Cuban, Salvadoran, Dominican, Columbian); Middle Eastern or North African (e.g., Lebanese, Iranian, Egyptian, Syrian, Moroccan, Algerian); Native Hawaiian or Other Pacific Islander (e.g., Native Hawaiian, Samoan, Chamorro, Tongan, Fijian, Marshallese); White (e.g., German, Irish, English, Italian, Polish, French); other; and prefer not to say. Due to the low frequencies of these groups in our sample, we categorized American Indian or Alaska Native, Middle Eastern or North African, Native Hawaiian or Other Pacific Islander, and other as “Other Race/ethnicity” for the analyses. 

##### Gender Identity

We included the following gender identity categories in the survey: cisgender male; cisgender female; transgender male; transgender female; non-binary/gender non-conforming; do not identify as female, male, or transgender; and prefer not to say. Our analysis grouped transgender, non-binary/gender non-conforming, and non-identifying individuals as “other gender identity” due to their low frequencies in the sample. 

##### Annual Household Income

Annual household income was operationalized as the following categories: less than USD 25,000, USD 25,000–34,999, USD 35,000–49,999, USD 50,000–74,999, USD 75,000–99,999, USD 100,000–149,999, USD 150,000–199,999, greater than or equal to USD 200,000, and prefer not to say. In the analysis, individuals with annual household incomes of USD 150,000 to USD 199,999 and USD 200,000 or more were grouped together due to their smaller frequencies in the sample.

##### Age

Age was a continuous variable that was provided as an open-ended response. We then created the following age categories: 18–25, 26–40, 41–64, and 65 and older. This operationalization was based on the rationale that individuals may have increased access to healthcare services until 26 (as they can stay on parental insurance), potentially increased need for healthcare after age 40 due to chronic illnesses and preventative screenings, and then changed access to healthcare after age 65 due to Medicare coverage. 

##### Marital Status

Marital status categories included: single/never married; married; not married, but in a relationship and living with your partner; not married, but in a relationship and not living with your partner; separated; divorced; widowed, and other. 

##### Region of Residence 

Respondents were asked to indicate the zip code of their residence in an open-ended response. These responses were then categorized into states, U.S. divisions, and then U.S. regions (Northeast, South, Midwest, and West) using the U.S. Census Bureau parameters [35].

##### Having Children under Age 18

Respondents were asked whether they had children under 18 years of age living with them; this variable was dichotomized as yes versus no. 

#### 2.2.3. Health-Related Variables

We investigated the following health-related correlates of access to health services:

##### Chronic Physical Health Condition

Chronic physical health condition was operationalized as a dichotomous variable (i.e., any versus none), indicating whether the respondent reported being diagnosed with at least one of the following chronic illnesses: multiple sclerosis, high blood pressure, COPD, diabetes, heart disease (heart failure, AFib, etc.), cancer, autoimmune (psoriatic disease, Crohn’s/ulcerative colitis, etc.), asthma, rheumatoid arthritis, and other.

##### Mental Health Condition

Mental health condition was also a dichotomous variable (any versus none); conditions included the following: mood disorder (e.g., depression, bipolar disorder, etc.), anxiety disorder (e.g., obsessive-compulsive disorder, panic disorder, phobias, etc.), eating disorder (e.g., anorexia, bulimia, etc.), post-traumatic stress disorder (PTSD), and other. 

##### Disability 

Disability was operationalized as a dichotomous variable (any versus none), indicating if the respondent reported having been diagnosed with any of the following disabilities: sensory impairment (vision or hearing), mobility impairment, learning disability (e.g., ADHD, dyslexia), and other. 

##### Health Insurance 

Health insurance was dichotomized as having any versus no health insurance. Health insurance categories included: private from employer, private from state health exchange, Medicaid, Medicare, and other. 

##### COVID-19 Diagnosis

Respondents were asked if they had been diagnosed with COVID-19 and if an immediate family member had been diagnosed with COVID-19. The “COVID-19 diagnosis” measure was dichotomized as any (i.e., if the respondent indicated “yes” to either of these questions) versus none (i.e., if the respondent indicated “no” to both questions). 

### 2.3. Analytical Approach 

**Figure 1:**
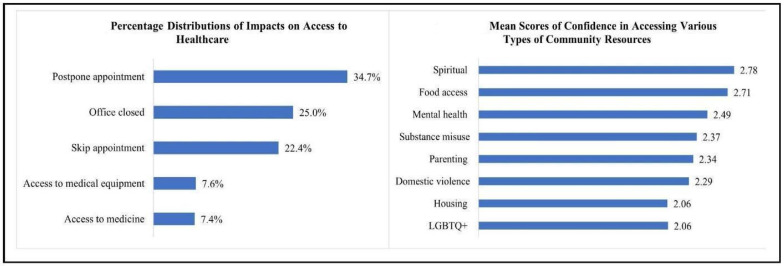
Access to Healthcare and Confidence in Accessing Community Resources during COVID-19 Pandemic.

The analyses were conducted using the StatsModels library in Python 3.7.13 [36]. Percentage distributions of each type of impact on access to healthcare (Figure 1) and mean scores for confidence in accessing each type of community resource were calculated. Linear regression models based on ordinary least squares (OLS) parameter estimation were then used to assess the relationships between sociodemographic- and health-related factors and access to health services (Table 3).

## 3. Results

### 3.1. Description of Sample

Table 3 contains the percentage distributions of the sample’s key sociodemographic and health characteristics. The sample was predominantly White (72%), followed by Black (12%), Asian (6%), multiracial (5%), and Hispanic, Latino, or Spanish origin (3%) individuals. Annual household income was relatively evenly distributed, with 25% having an income of less than USD 35,000 and approximately 27% with an income of more than USD 100,000. Regarding gender identity, 47% of the sample identified as cisgender female, 44% as cisgender male, and 1.9% as “other” (i.e., transgender; non-binary/non-gender conforming; or not identifying as male, female, or transgender). Almost half (45%) of the sample was married; 28% were single/never married; 13% were not married but in relationships, and 10% were divorced. Almost half (43%) of the sample was aged 41–64, followed in frequency by individuals aged 26 to 40 (27%), 18 to 25 (18%), and over 65 (12%). Further, 37% of the sample resided in the country’s Southern region, 21% in the West, 18% in the Midwest, and 14% in the Northeast. Only 29% of the sample reported having children under age 18.

Regarding the health characteristics of the sample, 46% of respondents reported having a chronic physical health condition; 35% had a mental health condition, and 20% had a disability. The majority (91%) of the sample reported having at least one type of health insurance. Further, 31% of respondents reported that they or an immediate family member had been diagnosed at least once with COVID-19.

Figure 1 illustrates participants’ access to healthcare and confidence in accessing community resources. Figure 1 shows how respondents’ access to various components of healthcare services was impacted during the pandemic. Postponing a healthcare appointment was the most common impact, with almost 35% of the sample citing it, followed by office closure (25%) and respondents needing to skip an appointment (22%). Impacts on access to healthcare visits were much more commonly reported than impacts on access to medicine and medical equipment during the pandemic. Figure 1 indicates that respondents expressed having the most confidence in accessing spiritual resources (mean = 2.78/4), followed by food access resources (mean = 2.71); they reported the lowest confidence scores in accessing housing resources (mean = 2.06) and LGBTQ+ resources (mean = 2.06). 

### 3.2. Multivariate Analysis 

Table 3 highlights the covariates that were statistically significant in the final multivariate models for each outcome variable, including impact on access to medicine and medical equipment, impact on access to healthcare visits, and confidence in accessing community resources. Individuals with an annual household income of USD 100,000–149,000 reported a significantly higher impact on their access to medicine and medical equipment when compared to individuals with incomes of USD 150,000 or more. Respondents who were not married but who were cohabiting with a partner reported significantly less impact on their access to medicine and medical equipment when compared to married respondents. Individuals aged 41 and older also experienced considerably less impact on this type of access compared to those aged 26–40. Further, those with at least one chronic physical health condition, a disability, a COVID-19 diagnosis in self or immediate family, and any children under age 18 reported significantly higher impact on their access to medicine and medical equipment than their counterparts.

In terms of access to healthcare visits, multiracial individuals experienced a significantly higher impact on access compared to White individuals (Table 3). Those in lower- and middle-income brackets (USD 35,000–49,999 and USD 75,000–99,999), as well as those who preferred not to report their incomes, experienced a significantly lower impact on their access to healthcare visits when compared to individuals with incomes of USD 150,000 or more. Further, cisgender females also experienced a significantly higher impact than cisgender males. Individuals aged 65 or older experienced significantly less impact on their access to healthcare visits compared to individuals aged 26–40. Additionally, respondents with any of the three pre-existing health conditions, health insurance, a COVID-19 diagnosis in self or immediate family, and any children under age 18 reported a higher impact on their access to healthcare visits. On the other hand, those who lived in the Midwest, South, and West reported significantly lower impact when compared to those living in the Northeast.

When examining differences related to confidence in accessing community resources, Asian individuals and individuals who were “other/prefer not to answer” reported significantly lower confidence when compared to White individuals (Table 3). Further, lower-income respondents (i.e., less than annual household incomes of USD 100,000) reported significantly less confidence in accessing community resources when compared to individuals with incomes of USD 150,000 or more. Individuals categorized as “other gender identity” (i.e., transgender, non-binary/gender non-conforming, and non-identifying individuals) reported significantly lower confidence in accessing resources than cisgender males. Finally, individuals aged 65 or older and those with health insurance had significantly higher confidence in accessing community resources when compared to those aged 26–40 and those without insurance, respectively.

## 4. Discussion

This study, which uses a large, nationwide sample, is among the first to provide nuanced insights into individuals’ access to health services during the pandemic. We developed and tested a new access-to-health-services measure, which included three key aspects: access to medicine and medical equipment, access to health visits, and access to community resources. When countries, including the U.S., made difficult decisions to balance the demands of responding directly to COVID-19 and maintaining essential health services, we observed with empirical data that individuals and communities not only struggled with scheduling and accessing healthcare visits, but access to medicine and supplies also became a critical aspect of healthcare accessibility. Problems, such as demand changes, regulation revisions, industry growth slow-down and pharmaceutical supply-chain disruptions, had short- and long-term impacts on the pharmaceutical sector [37,38] and the “call for action to advance equitable access to medicines” [39]. Quarantining and social isolation further challenged at-risk and vulnerable populations, highlighting the importance of community engagement and resources [40]. Community participation was critical in a pandemic for identifying solutions, circulating knowledge, providing insight into stigma and structural barriers, and devising collective responses [41]. Being able to access and leverage community resources, therefore, can be lifesaving. Our access-to-health-services measure contributes to the healthcare-access literature by encompassing and differentiating the three aspects of access to medicine and medical supplies, healthcare visits, and community resources.

Further, we found significant disparities in each of these outcome variables. Our findings support previous literature [2,6,42] that sociodemographic factors, such as race and gender, and health factors, such as chronic physical illness, mental health condition, or disability, were significant predictors of healthcare access disparities. By including access to community resources as an outcome variable, which has been understudied in existing research, we found that the influence of these sociodemographic factors spanned all three access domains. Further, the COVID-19 pandemic necessitated public health and social measures to reduce the spread of the disease, such as social isolation, quarantine, and school closure. These measures, however, led to disparities in access to health services that are related to social factors such as familial relationships, which were previously less researched. In our study, individuals who had children under 18 experienced some healthcare access challenges.

Additionally, in our study, individuals aged 65 or older reported significantly less impact on their healthcare access and higher confidence in accessing community resources. These Medicare-eligible individuals were perhaps more likely to be prioritized for healthcare access due to more pressing health concerns, and they may also have more experience with obtaining community resources due to age. Further, though we did not find consistent relationships between income and access to healthcare domains, our study revealed that those with lower annual household incomes reported lower confidence in accessing community resources. This finding corroborates evidence in the literature that, though they have a greater need for resources, poorer individuals often experience significantly reduced access to neighborhood and community resources compared to higher-income individuals [43].

Further, our study’s findings indicate the importance of broadening our understanding of cultural impacts on one’s access to healthcare and community resources. For instance, Asian and other minority groups reported lower confidence in accessing community resources crucial for their health and wellbeing during the pandemic. This resonates with prior literature [44] that states how social factors might impact ethnic minority families with a collectivistic cultural background very differently than White Americans or those from an individualistic cultural background. It is possible that increases in xenophobia and hate crimes directed at Asian Americans during the pandemic impacted this group’s comfort in seeking community-based resources [45].

The findings of this study have critical implications for programmatic and policy interventions. Beyond confirming existing knowledge, our study expands the concept of access to health services to include three major aspects: impact on access to medicine and medical equipment, impact on access to healthcare visits, and confidence in accessing community resources. In particular, we tested the instrument in the context of the COVID-19 pandemic, which showed the importance of accessing community resources during a public health crisis, a novel factor that was not considered explicitly in previous programs and policy making. These findings suggest that more effort should be placed on increasing the accessibility of community resources as a critical part of public health planning, both for preparing for public health emergencies and for achieving long-term public health outcomes. Further, this study helps expose how the healthcare system realistically functions during a global public health crisis; it informs the development of local, regional, and federal policies to ensure that vulnerable populations have equitable access to healthcare and community resources during future emergencies. Along these lines, this study sheds light on which populations are at the most significant risk for reduced access to healthcare and community services and therefore compels the development of programmatic interventions at the community and state levels to address the salient needs of these populations.

Overall, our study has several key strengths. Administered in August 2021 to a large, nationwide sample, the survey captures pandemic impacts at the national level over a long enough period to capture variability in accessing healthcare and community resources. Importantly, this study contributes to the literature by proposing a novel measurement of access to health services as well as simultaneously examining the multiple determinants of health, such as sociodemographic characteristics and health-related factors, and their relationships with the three access-to-health-services domains. Thus, we can gain a more comprehensive understanding of which individuals were most severely affected by the pandemic in terms of their access to healthcare and community resources. Additionally, our study’s findings revealed the complexity of the causes of disparities in access to health services. Shifting the discussion of access from a single-factor model to a comprehensive, multifaceted perspective that also incorporates the quality of care is necessary to ascertain the experiences, challenges, and barriers faced by individuals with diverse sociodemographic backgrounds and health characteristics. Along these lines, further research is needed to examine the potential mediating and moderating mechanisms by which sociodemographic and health characteristics may be related to the access-to-health-services measure. Finally, our study calls for longitudinal research to explore temporal relationships between reduced access to healthcare and community resources during the pandemic and longer-term health outcomes, such as the emergence of chronic physical and mental health issues.

### Limitations

Our study has some limitations that should be considered when interpreting its findings. First, as the survey was conducted in August 2021 and inquired about events that occurred in the past 17 months (since the lockdown in March 2020), responses may have been vulnerable to recall bias. Second, this study was cross-sectional. Longitudinal research using retrospective cohort design is needed to examine how this access may have differed during various stages of the pandemic and to explore causality between sociodemographic and health characteristics and access to healthcare and community resources. Third, there may have been potential selection bias due to the online administration of the survey. While 92% of U.S. residents have internet access [46], online recruitment may have precluded individuals with limited access or knowledge related to computers, smartphones, or internet access from participating in the study. Further, as individuals who lacked adequate access to the internet during the pandemic may also have been more severely affected by socioeconomic factors as well as the inability to access telemedicine, which was instrumental during this time period, our findings were likely conservative estimates of disparities in access to health services. Therefore, further research is needed to examine the unique challenges and experiences of this vulnerable population during the pandemic. Lastly, we acknowledge that data from online surveys is potentially more likely to contain missing data and be susceptible to survey fraud. However, we incorporated attention-check questions within the survey; respondents who failed these checks or had missing data were not included in our final sample.

## 5. Conclusions

The COVID-19 pandemic has impacted various aspects of people’s lives, including access to health services. It is known that race/ethnicity, gender, socioeconomic status, age, sexual orientation, and gender identity are significant predictors of disparities in healthcare access [2,6,7,8,9,10,11]. There is, however, limited research on disparities in access to community resources and how the COVID-19 pandemic may have impacted these differences. This study contributes to the growing literature on the multidimensional impacts of the pandemic by developing and testing a new access-to-health-services measure, comprising access to medicine and medical equipment, healthcare visits, and community resources and examining disparities in access to health services among individuals in the U.S. during the COVID-19 pandemic. Our study’s findings indicate that disparities in access to healthcare during the pandemic were associated with sociodemographic characteristics, such as race, gender, and age, and health-related characteristics, such as chronic illness, mental health condition, and disability. Familial factors, such as having children under the age of 18, made individuals more vulnerable to experiencing reduced access to healthcare as well. Additionally, we found that factors such as race, gender, income, and age were associated with individuals’ degree of confidence in accessing community services. These findings have critical implications for the creation of programmatic interventions to address the long-term needs of populations whose access to health services was most impacted during the pandemic as well as the development of policies to better prepare for future global health emergencies. Further longitudinal research is needed to explore the temporal relationships between decreased access to healthcare and community resources during the pandemic and potential increases in mortality and morbidity in the population.

## Figures and Tables

**Table 1:** Factor analysis of the access to healthcare measures.

Factors	Items	Components	
		1	2
Access to Visits	Could not go to an appointment because the office was closed	0.644	0.221
	Had to postpone a healthcare appointment that you have rescheduled or intend to reschedule	0.802	−0.030
	Had to skip a healthcare appointment	0.670	0.116
Access to Medicine and Equipment	Could not obtain necessary equipment or supplies	0.092	0.804
	Could not obtain necessary medications	0.068	0.798
Rotation Sum of Squared Loadings	Total	2.122	1.104
	% Variance	35.36	18.394
	Cumulative Variance	35.362	53.756

**Table 2:** Components of access-to-health-services measure.

Components and Items	Range of Scores	Cronbach’s Alpha
Access to healthcare		
*Access to medicine and medical equipment*	0–2	0.65
Could not obtain necessary equipment or supplies	0–1	
Could not obtain necessary medications	0–1	
*Access to healthcare visits*	0–3	0.66
Could not go to an appointment because the office was closed	0–1	
Had to postpone a healthcare appointment that you have rescheduled or intend to reschedule	0–1	
Had to skip a healthcare appointment	0–1	
Access to community resources		
*Confidence in accessing following community resources:*	0–32	0.89
Food access support	0–4	
Housing support	0–4	
Domestic violence resources	0–4	
Mental health resources	0–4	
Substance misuse resources	0–4	
Parenting/family support services	0–4	
LGBTQ+ support	0–4	
Spiritual/religious resources	0–4	

**Table 3:** Sociodemographic- and health-related correlates of access to health services.

	Impact on Access to Medicine and Medical Equipment		Impact on Access to Healthcare Visits		Confidence in Accessing Community Resources	
	β (SE)	*p* ^a^	β (SE)	*p* ^a^	β (SE)	*p* ^a^
**n = 1491**	Range = 0–2 Mean (SD) = 0.15 (0.44) R^2^ = 0.111		Range 0–3 Mean (SD) = 0.82 (0.97) R^2^ = 0.090		Range 0–32 Mean (SD) = 19.10 (7.21) R^2^ = 0.060	
**Sociodemographic factors**						
Race/ethnicity						
Asian (5.8%)	0.04 (0.05)	0.463	0.04 (0.11)	0.725	1.66 (0.83)	**0.046**
Black or African American (12.2%)	0.04 (0.04)	0.302	0.12 (0.08)	0.135	0.56 (0.60)	0.355
Hispanic, Latino, or Spanish Origin (3.4%)	0.01 (0.06)	0.927	0.01 (0.14)	0.961	1.14 (1.05)	0.278
Multiracial (4.8%)	0.04 (0.05)	0.455	0.29 (0.12)	**0.013**	0.57 (0.88)	0.517
White (72.4%)	ref	--	ref	--	ref	--
Other/Prefer not to say (1.3%)	0.18 (0.10)	0.058	0.13 (0.21)	0.556	3.70 (1.62)	**0.023**
Annual household income						
Less than USD 25,000 (16.2%)	0.07 (0.05)	0.144	0.14 (0.10)	0.167	2.04 (0.79)	**0.010**
USD 25,000 to 34,999 (8.9%)	0.05 (0.05)	0.372	0.13 (0.12)	0.262	2.75 (0.88)	**0.002**
USD 35,000 to 49,999 (13.9%)	0.02 (0.05)	0.647	0.26 (0.10)	**0.012**	1.78 (0.79)	**0.024**
USD 50,000 to 74,999 (16.6%)	0.01 (0.04)	0.746	0.14 (0.10)	0.154	1.48 (0.75)	**0.047**
USD 75,000 to 99,999 (14.0%)	0.01 (0.05)	0.830	0.20 (0.10)	**0.046**	1.20 (0.76)	0.117
USD 100,000 to 149,999 (16.8%)	0.10 (0.04)	**0.024**	0.17 (0.10)	0.090	0.74 (0.73)	0.314
USD 150,000 or more (10.5%)	ref	--	ref	--	ref	--
Prefer not to say (3.2%)	0.02 (0.07)	0.790	0.37 (0.16)	**0.024**	1.09 (1.23)	0.376
Gender identity						
Cisgender female (47.1%)	0.05 (0.02)	0.059	0.14 (0.05)	**0.008**	0.45 (0.41)	0.267
Cisgender male (44.4%)	ref	--	ref	--	ref	--
Other gender identity (1.9%)	0.10 (0.08)	0.228	0.13 (0.18)	0.496	2.75 (1.39)	**0.048**
Prefer not to say (6.6%)	0.04 (0.05)	0.440	0.11 (0.11)	0.295	0.19 (0.79)	0.811
Marital status						
Divorced (9.9%)	0.04 (0.04)	0.347	0.02 (0.09)	0.840	0.21 (0.70)	0.766
Married (45.4%)	ref	--	ref	--	ref	--
Not married, but in a relationship and living together (7.6%)	0.10 (0.05)	**0.038**	0.13 (0.10)	0.197	0.26 (0.78)	0.736
Not married, but in a relationship and not living together (5.6%)	0.10 (0.06)	0.070	0.10 (0.13)	0.449	0.03 (0.95)	0.978
Separated (0.8%)	0.22 (0.12)	0.078	0.07 (0.28)	0.790	1.63 (2.08)	0.433
Single/never married (27.6%)	0.07 (0.03)	0.055	0.10 (0.08)	0.186	0.01 (0.58)	0.987
Widowed (2.1%)	0.01 (0.08)	0.872	0.22 (0.17)	0.206	2.62 (1.31)	**0.046**
Unknown or prefer not to say (0.9%)	0.00 (0.12)	0.983	0.28 (0.26)	0.275	4.92 (1.94)	**0.012**
Age						
18 to 25 (18.2%)	0.01 (0.04)	0.713	0.06 (0.09)	0.492	0.68 (0.65)	0.298
26 to 40 (26.8%)	ref	--	ref	--	ref	--
41 to 64 (43.0)	0.06 (0.03)	**0.037**	0.02 (0.07)	0.825	0.54 (0.50)	0.277
65 or older (11.9%)	0.17 (0.04)	**<0.001**	0.24 (0.10)	**0.015**	1.58 (0.74)	**0.032**
Region of residence						
Midwest (18.0%)	0.03 (0.04)	0.509	0.23 (0.09)	**0.008**	0.30 (0.66)	0.647
Northeast (14.4%)	ref	--	ref	--	ref	--
South (37.0%)	0.05 (0.03)	0.136	0.22 (0.08)	**0.004**	0.22 (0.58)	0.710
West (20.7%)	0.07 (0.04)	0.067	0.15 (0.08)	0.072	0.19 (0.64)	0.769
Prefer not to say (9.9%)	0.03 (0.05)	0.552	0.16 (0.10)	0.122	0.19 (0.78)	0.803
Any children under age 18						
Yes (29.0%)	0.17 (0.03)	**<0.001**	0.20 (0.06)	**0.002**	0.77 (0.49)	0.112
No (71.0%)	ref	--	ref	--	ref	--
**Pre-existing health conditions**						
Any chronic physical health condition						
Yes (45.9%)	0.08 (0.02)	**0.001**	0.22 (0.05)	**<0.001**	0.38 (0.41)	0.348
No (54.1%)	ref	--	ref	--	ref	--
Any mental health condition						
Yes (34.9%)	0.01 (0.02)	0.593	0.17 (0.06)	**0.002**	0.32 (0.42)	0.434
No (65.1%)	ref	--	ref	--	ref	--
Any disability						
Yes (20.2%)	0.08 (0.03)	**0.007**	0.15 (0.06)	**0.021**	0.09 (0.48)	0.858
No (79.8%)	ref	--	ref	--	ref	--
Any health insurance						
Yes (91.0%)	0.02 (0.04)	0.547	0.29 (0.09)	**0.001**	1.39 (0.68)	**0.040**
No (9.0%)	ref	--	ref	--	ref	--
COVID-19 diagnosis in self or family						
Yes (30.8%)	0.07 (0.02)	**0.003**	0.12 (0.05)	**0.026**	0.11 (0.41)	0.797
No (69.2%)	ref	--	ref	--	ref	--

ref = reference group; ^a^ Bolded *p*-values are at <0.05 significance levels.

## Data Availability

The data supporting this study’s findings are available from the corresponding author upon reasonable request.

## References

[1] Rice T., Rosenau P., Unruh L.Y., Barnes A.J., Saltman R.B., van Ginneken E. (2013). United States of America: Health System Review. Health Syst. Transit..

[2] Waidmann T.A., Rajan S. (2000). Race and Ethnic Disparities in Health Care Access and Utilization: An Examination of State Variation. Med. Care Res. Rev..

[3] Gebo K.A., Fleishman J.A., Conviser R., Reilly E.D., Korthuis P.T., Moore R.D., Hellinger J., Keiser P., Rubin H.R., Crane L. (2005). Racial and Gender Disparities in Receipt of Highly Active Antiretroviral Therapy Persist in a Multistate Sample of HIV Patients in 2001. JAIDS J. Acquir. Immune Defic. Syndr..

[4] Mehta N.K., Lee H., Ylitalo K.R. (2013). Child Health in the United States: Recent Trends in Racial/Ethnic Disparities. Soc. Sci. Med..

[5] Jarlenski M., Baller J., Borrero S., Bennett W.L. (2016). Trends in Disparities in Low-Income Children’s Health Insurance Coverage and Access to Care by Family Immigration Status. Acad. Pediatr..

[6] Pederson A., Raphael D., Johnson E. (2016). Gender, Race, and Health Inequalities. Rethinking Society in the 21 Century: Critical Readings in Sociology.

[7] Adamson J., Ben-Shlomo Y., Chaturvedi N., Donovan J. (2003). Ethnicity, Socio-Economic Position and Gender—Do They Affect Reported Health—Care Seeking Behaviour?. Soc. Sci. Med..

[8] Shavers V.L. (2007). Measurement of Socioeconomic Status in Health Disparities Research. J. Natl. Med. Assoc..

[9] Read J.G., Gorman B.K. (2006). Gender Inequalities in US Adult Health: The Interplay of Race and Ethnicity. Soc. Sci. Med..

[10] Dunlop D.D., Manheim L.M., Song J., Chang R.W. (2002). Gender and Ethnic/Racial Disparities in Health Care Utilization Among Older Adults. J. Gerontol. B Psychol. Sci. Soc. Sci..

[11] Yeung H., Luk K.M., Chen S.C., Ginsberg B.A., Katz K.A. (2019). Dermatologic Care for Lesbian, Gay, Bisexual, and Transgender Persons. J. Am. Acad. Dermatol..

[12] Quinn S.C., Kumar S., Freimuth V.S., Musa D., Casteneda-Angarita N., Kidwell K. (2011). Racial Disparities in Exposure, Susceptibility, and Access to Health Care in the US H1N1 Influenza Pandemic. Am. J. Public Health.

[13] Chotiner I. (2020). The Interwoven Threads of Inequality and Health. The New Yorker.

[14] Eberly L.A., Kallan M.J., Julien H.M., Haynes N., Khatana S.A.M., Nathan A.S., Snider C., Chokshi N.P., Eneanya N.D., Takvorian S.U. (2020). Patient Characteristics Associated With Telemedicine Access for Primary and Specialty Ambulatory Care During the COVID-19 Pandemic. JAMA Netw. Open.

[15] Gmunder K.N., Ruiz J.W., Franceschi D., Suarez M.M. (2021). Demographics Associated with US Healthcare Disparities Are Exacerbated by the Telemedicine Surge during the COVID-19 Pandemic. J. Telemed. Telecare.

[16] Institute of Medicine (U.S.) (1993). Committee on Monitoring Access to Personal Health Care Services Access to Health Care in America.

[17] Mooney G., Hall J., Donaldson C., Gerard K. (1991). Utilisation as a Measure of Equity: Weighing Heat?. J. Health Econ..

[18] Culyer A.J., van Doorslaer E., Wagstaff A. (1992). Access, Utilisation and Equity: A Further Comment. J. Health Econ..

[19] Peters D.H., Garg A., Bloom G., Walker D.G., Brieger W.R., Hafizur Rahman M. (2008). Poverty and Access to Health Care in Developing Countries. Ann. N. Y. Acad. Sci..

[20] World Health Organization (2020). Pulse Survey on Continuity of Essential Health Services during the COVID-19 Pandemic.

[21] Magadzire B.P., Budden A., Ward K., Jeffery R., Sanders D. (2014). Frontline Health Workers as Brokers: Provider Perceptions, Experiences and Mitigating Strategies to Improve Access to Essential Medicines in South Africa. BMC Health Serv. Res..

[22] Yaghoubifard S., Rashidian A., Kebriaeezadeh A., Majdzadeh R., Hosseini S.A., Sari A.A., Salamzadeh J. (2015). Developing a Conceptual Framework and a Tool for Measuring Access to, and Use of, Medicines at Household Level (HH-ATM Tool). Public Health.

[23] Artiga S., Hinton E. (2018). Beyond Health Care: The Role of Social Determinants in Promoting Health and Health Equity. Kaiser Family Foundation. https://www.kff.org/racial-equity-and-health-policy/issue-brief/beyond-health-care-the-role-of-social-determinants-in-promoting-health-and-health-equity/.

[24] Kurtzleben D. (2020). Job Losses Higher Among People of Color During Coronavirus Pandemic. NPR.

[25] Lopez L., Hart L.H., Katz M.H. (2021). Racial and Ethnic Health Disparities Related to COVID-19. JAMA.

[26] Prolific. https://www.prolific.co.

[27] Peer E., Rothschild D., Gordon A., Evernden Z., Damer E. (2021). Data Quality of Platforms and Panels for Online Behavioral Research. Behav. Res. Methods.

[28] Newman A., Bavik Y.L., Mount M., Shao B. (2021). Data Collection via Online Platforms: Challenges and Recommendations for Future Research. Appl. Psychol..

[29] Palan S., Schitter C. (2018). Prolific.Ac—A Subject Pool for Online Experiments. J. Behav. Exp. Finance.

[30] Tang J., Birrell E., Lerner A. (2022). Replication: How Well Do My Results Generalize Now? The External Validity of Online Privacy and Security Surveys.

[31] Núñez A., Sreeganga S.D., Ramaprasad A. (2021). Access to Healthcare during COVID-19. Int. J. Environ. Res. Public. Health.

[32] Kaye A.D., Okeagu C.N., Pham A.D., Silva R.A., Hurley J.J., Arron B.L., Sarfraz N., Lee H.N., Ghali G.E., Gamble J.W. (2021). Economic Impact of COVID-19 Pandemic on Healthcare Facilities and Systems: International Perspectives. Best Pract. Res. Clin. Anaesthesiol..

[33] Vaccaro A.R., Getz C.L., Cohen B.E., Cole B.J., Donnally C.J. (2020). Practice Management during the COVID-19 Pandemic. J. Am. Acad. Orthop. Surg..

[34] Ahmed T., Lodhi S.H., Kapadia S., Shah G.V. (2020). Community and Healthcare System-Related Factors Feeding the Phenomenon of Evading Medical Attention for Time-Dependent Emergencies during COVID-19 Crisis. BMJ Case Rep..

[35] U.S. Census Bureau Census Regions and Divisions of the United States. https://www2.census.gov/geo/pdfs/maps-data/maps/reference/us_regdiv.pdf.

[36] Python StatsModels Library 2022. www.statsmodels.org.

[37] Alexander G.C., Qato D.M. (2020). Ensuring Access to Medications in the US During the COVID-19 Pandemic. JAMA.

[38] Ayati N., Saiyarsarai P., Nikfar S. (2020). Short and Long Term Impacts of COVID-19 on the Pharmaceutical Sector. DARU J. Pharm. Sci..

[39] Kohler J.C., Mackey T.K. (2020). Why the COVID-19 Pandemic Should Be a Call for Action to Advance Equitable Access to Medicines. BMC Med..

[40] Evans M.L., Lindauer M., Farrell M.E. (2020). A Pandemic within a Pandemic—Intimate Partner Violence during COVID-19. N. Engl. J. Med..

[41] Marston C., Renedo A., Miles S. (2020). Community Participation Is Crucial in a Pandemic. Lancet.

[42] Shi L., Chen C.-C., Nie X., Zhu J., Hu R. (2014). Racial and Socioeconomic Disparities in Access to Primary Care Among People With Chronic Conditions. J. Am. Board Fam. Med..

[43] Pearce J., Witten K., Hiscock R., Blakely T. (2007). Are Socially Disadvantaged Neighbourhoods Deprived of Health-Related Community Resources?. Int. J. Epidemiol..

[44] Hwang W.-C. (2016). Culturally Adapting Psychotherapy for Asian Heritage Populations: An Evidence-Based Approach.

[45] Li Y., Galea S. (2020). Racism and the COVID-19 Epidemic: Recommendations for Health Care Workers. Am. J. Public Health.

[46] Statistica Internet User Penetration in the United States from 2018 to 2027. https://www.statista.com/statistics/590800/internet-usage-reach-usa/.

